# Higher Cardiorespiratory Fitness Is Associated With a Lower Burden of Metabolic Syndrome Traits: A Cross‐Sectional Analysis of 39 197 UK Adults

**DOI:** 10.1111/dom.71367

**Published:** 2026-09-17

**Authors:** Scott A. Willis, Annalena Phillipp, Kevin Deighton, Christopher Goodwin, Daniel J. Cuthbertson, Shelley E. Keating, Christopher J. A. Pugh, Jonathan G. Stine, Gaël Ennequin, Alexis Couret, David J. Stensel, Guruprasad P. Aithal, Thomas Yates, James A. King

**Affiliations:** ^1^ National Centre for Sport and Exercise Medicine, School of Sport, Exercise and Health Sciences Loughborough University Loughborough UK; ^2^ NIHR Leicester Biomedical Research Centre University Hospitals of Leicester NHS Trust and the University of Leicester Leicester UK; ^3^ Diabetes Research Centre, College of Life Sciences University of Leicester Leicester UK; ^4^ Department for Physical Activity in Public Health Institute of Movement and Neurosciences, German Sport University Cologne Cologne Germany; ^5^ Nuffield Health Research Group Nuffield Health Epsom UK; ^6^ Department of Cardiovascular and Metabolic Medicine Institute of Life Course and Medical Sciences, University of Liverpool Liverpool UK; ^7^ Liverpool University Hospitals NHS Foundation Trust Liverpool UK; ^8^ School of Human Movement and Nutrition Sciences The University of Queensland St Lucia Queensland Australia; ^9^ School of Life Course & Population Health Sciences King's College London London UK; ^10^ Division of Gastroenterology and Hepatology, Department of Medicine Penn State Health Milton S. Hershey Medical Center Hershey Pennsylvania USA; ^11^ Laboratory of the Metabolic Adaptations to Exercise Under Physiological and Pathological Conditions (AME2P) Clermont Auvergne University Clermont‐Ferrand France; ^12^ International Research Chair Health in Motion Clermont Auvergne University Foundation Clermont‐Ferrand France; ^13^ Faculty of Sport Sciences Waseda University Tokorozawa Japan; ^14^ Nottingham Digestive Diseases Centre, Translational Medical Sciences, School of Medicine University of Nottingham Nottingham UK; ^15^ NIHR Nottingham Biomedical Research Centre Nottingham University Hospitals NHS Trust and the University of Nottingham Nottingham UK

**Keywords:** disease prevention, dose–response relationship, dyslipidaemia, obesity care, observational study, population study

## Abstract

**Aims:**

To investigate associations of cardiorespiratory fitness (CRF) with metabolic syndrome (MetS) trait count, MetS prevalence and individual MetS traits in a large population‐based cohort.

**Materials and Methods:**

This cross‐sectional study analysed data for 39 197 adults from a UK‐based independent healthcare charity (mean ± SD: age, 45.8 ± 9.0 years; BMI, 26.3 ± 4.1 kg⋅m^−2^; 31% female). CRF was estimated as peak oxygen uptake from submaximal treadmill or cycle ergometer protocols and analysed continuously and as sex‐ and mode‐specific quintiles. MetS was defined using established criteria, with outcomes examined as trait count (0–5), presence (≥ 3 traits) and individual traits. Associations were assessed using negative binomial regression for trait count and Poisson regression for MetS presence. Models were sequentially adjusted for demographics (primary model), adiposity (BMI) and hepatic steatosis (fatty liver index [FLI]).

**Results:**

Each 1 mL⋅kg^−1^⋅min^−1^ higher CRF was associated with a 2.9% lower MetS trait count and 6.0% lower MetS prevalence (*p* < 0.001). Compared with the highest CRF quintile, the lowest quintile had a 2.4‐fold higher MetS trait count and 5.4‐fold higher MetS prevalence (*p* < 0.001). Associations persisted after BMI and FLI adjustment, although attenuated. Spline analyses demonstrated graded, non‐linear inverse associations, steepest at lower CRF levels. Higher CRF was inversely associated with all individual MetS traits, strongest for central obesity.

**Conclusions:**

Higher CRF was associated with lower metabolic risk burden and MetS prevalence, which remained after adjustment for adiposity‐related measures. These findings support the potential public health relevance of modest fitness improvements, particularly among the least fit adults, as a strategy that may help reduce MetS burden even when adiposity changes are limited.

## 1 Introduction

The metabolic syndrome (MetS) represents a clustering of cardiometabolic abnormalities, including central obesity, hypertension, hyperglycaemia, elevated triglycerides and low high‐density lipoprotein cholesterol (HDL‐c) [1]. The co‐occurrence of these abnormalities is associated with increased risk of cardiovascular disease (CVD), type 2 diabetes, metabolic dysfunction‐associated steatotic liver disease (MASLD), certain cancers and all‐cause mortality [2, 3, 4]. Recent global estimates suggest that 1.54 billion people are living with MetS, with prevalence having doubled in women and nearly tripled in men over the last 25 years [5]. These data highlight the growing clinical and public health importance of identifying modifiable determinants of metabolic risk.

Recent research has moved beyond the traditional dichotomous definition of MetS by operationalising severity according to the number of abnormal MetS traits present. MetS trait count captures the graded nature of metabolic risk, recognising that adverse clinical outcomes increase incrementally as cardiometabolic abnormalities accumulate. For example, in individuals with MASLD, higher MetS trait count dose‐dependently predicts major adverse liver outcomes, CVD, chronic kidney disease and obesity‐related cancers [2, 6, 7, 8]. Compared with a binary construct, trait count may provide more granular risk stratification while remaining clinically interpretable and more intuitive than continuous MetS *Z*‐score approaches [2]. However, despite its potential clinical utility, little is known about how modifiable movement‐related factors, including cardiorespiratory fitness (CRF), relate to the accumulation of MetS traits.

CRF is an objectively quantifiable and clinically important indicator of whole‐body physiological function, reflecting the integrated capacity of the cardiovascular, respiratory and musculoskeletal systems, shaped by habitual physical activity and genetic constitution [9]. It can be measured directly or estimated using exercise‐ or non‐exercise‐based prediction equations. Higher CRF has been consistently associated with lower prevalence and incidence of MetS [10, 11, 12, 13, 14, 15, 16, 17, 18], with associations appearing partly independent of body composition, smoking and other sociodemographic and clinical factors [12, 15, 18, 19]. However, the relationship between CRF and MetS trait burden is less well characterised. Although previous studies suggest that lower CRF may be associated with more MetS components, these were limited by small samples, male‐only populations and limited examination of individual traits [12, 20, 21]. Thus, the extent to which CRF relates to MetS trait accumulation, and whether these relationships differ across individual traits or are independent of adiposity, remains unclear. Clarifying these relationships may inform more targeted exercise prescription and risk‐reduction strategies for individuals with differing levels of metabolic dysfunction.

Accordingly, the present study analysed a large, population‐based cohort of 39 197 UK adults, including males and females, with an exercise‐based estimate of CRF. The primary aim was to investigate the relationship between CRF and MetS trait count. Secondary aims were to determine the extent to which these associations were attenuated after adjustment for total and liver‐specific adiposity, and to characterise associations between CRF and individual MetS traits.

## 2 Methods

### 2.1 Study Design and Participants

In this cross‐sectional study, anonymised data were analysed from standardised health assessments conducted by a UK healthcare charity between 2006 and 2024. Full cohort details have been described previously [22, 23]. The dataset comprised adults aged 18–83 years who voluntarily attended health assessments at hospitals, fitness centres and corporate locations across the UK for a variety of reasons including for a general health check, medical concern or occupational requirement. All assessment locations included participants attending for a combination of these reasons. No additional study‐specific inclusion or exclusion criteria were applied; participants were included in the present analyses where sufficient data were available to derive the relevant exposure, outcome and covariate variables.

Assessments included anthropometry, CRF, blood pressure and cardiometabolic biomarkers. Before assessment, participants completed a questionnaire capturing demographic information and health characteristics.

Ethical approval was obtained from the Loughborough University Ethics Review Sub‐Committee (project ID: 15414) and participants consented for their anonymised data to be used for research. Reporting follows the Strengthening the Reporting of Observational Studies in Epidemiology (STROBE) guidelines for cross‐sectional studies [24], and the study was conducted in accordance with the Declaration of Helsinki (2013).

### 2.2 Standardised Health Assessments

Assessments were performed by a medical doctor and/or health and wellbeing physiologist. Height was measured using a stadiometer to the nearest 0.1 cm and body mass using calibrated digital scales to the nearest 0.1 kg. Body mass index (BMI; kg⋅m^−2^) was calculated from measured height and body mass. Waist circumference was measured to the nearest 0.1 cm using a non‐elastic tape at the midpoint between the lower rib and iliac crest. Body fat percentage was estimated using bioelectrical impedance analysis using a commercially available analyser. Resting systolic blood pressure (SBP) and diastolic blood pressure (DBP) were measured using an automated sphygmomanometer following European Society of Hypertension recommendations [25].

### 2.3 CRF Assessment (Exposure)

A standardised graded exercise test estimated participants' CRF. CRF was defined as the estimated peak oxygen uptake corresponding to the highest exercise level attained during the assessment, expressed in mL⋅kg^−1^⋅min^−1^. Testing was performed on either a treadmill or cycle ergometer depending on equipment availability at the assessment centre or participant preference where both modalities were available. Standardised protocols were used consistently across assessment centres for each exercise modality. Treadmill assessments were based on the Bruce protocol [26], with estimated VO_2_peak derived from total exercise duration using established sex‐specific equations [27, 28]. Cycle ergometer assessments were based on an established staged ramp protocol with progressive workload increases every 2 min [29, 30]; estimated VO_2_peak was derived from final power output (Watts) and body mass using sex‐specific equations [31, 32]. These protocols align with American College of Sports Medicine (ACSM) recommendations [33] and are widely used in clinical and population‐based health assessments [34]. Importantly, given the nature of the healthcare assessments and established safety procedures, both protocols were modified to be submaximal, with testing terminated at the earliest occurrence of 85% of age‐predicted maximal heart rate, fatigue, clinical indication or other termination criteria. Accordingly, derived values represent estimated peak VO_2_ during the test rather than directly measured maximal VO_2_. Additional methodological details are provided in the Supporting Information Methods. For categorical analyses, CRF was divided into quintiles constructed separately by sex and exercise test modality.

### 2.4 Blood Sampling and Biochemical Measures

Venous blood samples were collected following an overnight fast and processed by local pathology laboratories. Samples were refrigerated (4°C) after collection and analysed within 24 h. Biochemical measurements included triglycerides, HDL‐c, fasting glucose and gamma‐glutamyl transferase (GGT) [35].

### 2.5 MetS Traits (Outcomes)

Five individual MetS traits were evaluated: central obesity, hypertension, hyperglycaemia, low HDL‐c and elevated triglycerides. Each component was defined using standard established thresholds. Central obesity was defined as a waist circumference ≥ 94 cm in males and ≥ 80 cm in females, while hypertension was defined as SBP ≥ 130 mmHg or DBP ≥ 85 mmHg. Hyperglycaemia was defined as fasting glucose concentrations ≥ 5.6 mmol⋅L^−1^, while low HDL‐c was defined as HDL‐c concentrations < 1.0 mmol⋅L^−1^ in males and < 1.3 mmol⋅L^−1^ in females. Elevated triglycerides were classified as triglyceride concentrations ≥ 1.7 mmol⋅L^−1^. MetS trait count was calculated by summing abnormal traits present (range 0–5). Established MetS was defined as three or more traits, consistent with commonly used diagnostic criteria [1].

### 2.6 Covariates

Covariates included age, sex, socioeconomic status (SES), calendar year of assessment, UK region of assessment centre, CRF assessment mode, BMI and fatty liver index (FLI).

Age was calculated as the time between date‐of‐birth and date of health assessment, while sex was self‐reported as male or female. SES was determined using the national Index of multiple deprivation corresponding to each participant's country of residence. Scores were harmonised by allocating individuals to deprivation deciles (1 = most deprived; 10 = least deprived) according to residential postcode. UK region of assessment centre was categorised into 11 regions (nine English regions plus Scotland and Wales).

BMI was used as a marker of total adiposity, while FLI was used as a marker of liver‐specific adiposity. FLI was calculated from BMI, waist circumference, triglycerides and GGT using established equations [36].

### 2.7 Statistical Analysis

Statistical analyses were performed using SPSS Statistics (version 31; SPSS Inc., Chicago, Illinois), while visual presentation used GraphPad Prism (version 11; GraphPad Software, San Diego, California) and R (version 4.5.1; R Foundation for Statistical Computing, Vienna, Austria). The distribution of continuous variables was assessed using Kolmogorov–Smirnov tests and visual inspection of histograms. Normally distributed continuous variables are presented as means ± standard deviations (SD), non‐normally distributed variables as medians (interquartile ranges), and categorical variables as frequencies [percentages].

Associations between CRF and MetS trait count were examined using negative binomial regression, due to overdispersion in the outcome distribution, and are reported as rate ratios (RRs) with 95% confidence intervals (CIs). Associations between CRF and MetS presence, and each individual MetS component, were examined using Poisson regression with robust standard errors, appropriate for binary outcomes with non‐rare prevalence, and are expressed as prevalence ratios (PRs) with 95% CIs. CRF was analysed continuously, with effect estimates expressed per 1 mL⋅kg^−1^⋅min^−1^ increase, and as sex‐ and mode‐specific quintiles, with the highest fitness quintile (Q5) as the reference.

Two primary models were constructed. Model 1 included minimal adjustment for CRF assessment mode (binary). Model 2 (demographic‐adjusted) additionally adjusted for age (continuous), sex (binary), SES (ordinal), assessment year (continuous) and UK region of assessment centre (categorical). To explore the extent to which associations were attenuated after accounting for adiposity and hepatic steatosis, two additional models were fitted. Model 3 (adiposity‐adjusted) additionally adjusted for BMI (continuous), and Model 4 (hepatic steatosis‐adjusted) additionally adjusted for both BMI and FLI (continuous). Given that FLI incorporates waist circumference and triglycerides, which are also components of the MetS outcome, Model 4 was considered exploratory. Sensitivity analyses repeated Models 3 and 4, substituting body fat percentage for BMI to evaluate robustness of adiposity adjustments.

To assess and visualise non‐linear associations, natural cubic splines (four degrees of freedom) were incorporated into the demographic‐adjusted model (Model 2), modelling CRF continuously. Marginal predictions of MetS trait count and MetS prevalence were generated across a pre‐specified CRF range (15–70 mL⋅kg^−1^⋅min^−1^) using Poisson regression with robust standard errors (selected for computational stability over negative binomial regression in spline estimation). Predicted values represent covariate‐adjusted estimates calculated at fixed values of confounders (median age, SES and assessment year; and the most frequent categories of CRF assessment mode and UK region). Sex was fixed separately at male or female to produce sex‐specific spline curves and evaluate potential heterogeneity.

All results are reported with 95% CIs and exact *p*‐values. Effect estimates > 1.000 indicate higher MetS burden or prevalence. Statistical significance was defined as *p* < 0.05.

## 3 Results

### 3.1 Participant Characteristics

Overall, 39 197 participants with complete data for the main analyses were included. Figure S1 summarises participant flow and reasons for exclusion, including non‐fasted assessments, missing study variables and implausible values. Characteristics of the included analytic cohort are presented in Table 1, while characteristics of participants included in and excluded from the main analyses are compared in Table S1.

**TABLE 1:** Participant characteristics of the total study cohort and stratified by cardiorespiratory fitness quintile.

Variable	Total (*n* = 39 197)	Q1 (*n* = 7815) (lowest CRF)	Q2 (*n* = 7763)	Q3 (*n* = 7950)	Q4 (*n* = 8114)	Q5 (*n* = 7555) (highest CRF)
Demographics						
Age (years)	45.8 ± 9.0	49.3 ± 8.9	47.0 ± 8.6	46.0 ± 8.4	44.4 ± 8.6	42.4 ± 9.1
Sex						
Female [%]	12 331 [31.5]	2410 [30.8]	2422 [31.2]	2575 [32.4]	2552 [31.5]	2372 [31.4]
Male [%]	26 866 [68.5]	5405 [69.2]	5341 [68.8]	5375 [67.6]	5562 [68.5]	5183 [68.6]
SES (decile)	8 (6–9)	8 (6–9)	8 (6–10)	8 (6–9)	8 (6–10)	8 (6–9)
Adiposity and body composition						
Weight (kg)	80.7 ± 15.7	87.9 ± 18.0	83.1 ± 15.4	80.7 ± 14.0	77.5 ± 13.4	74.5 ± 13.6
BMI (kg⋅m^−2^)	26.3 ± 4.1	28.8 ± 4.8	27.0 ± 3.9	26.3 ± 3.4	25.3 ± 3.2	24.3 ± 3.2
Body fat (%)	25.9 ± 7.5	29.8 ± 7.7	27.2 ± 7.2	26.2 ± 7.2	24.2 ± 6.7	22.2 ± 6.5
Fatty liver index (AU)	30.1 (11.5–60.2)	57.0 (26.3–81.9)	41.6 (17.2–68.7)	32.0 (14.3–57.2)	21.3 (9.4–43.2)	13.6 (6.1–31.6)
Cardiorespiratory fitness						
CRF (mL⋅kg^−1^⋅min^−1^)	37.2 ± 10.0	25.6 ± 3.9	32.1 ± 3.3	36.5 ± 3.7	41.3 ± 4.4	51.0 ± 9.3
CRF, cycle ergometer (mL⋅kg^−1^⋅min^−1^)	35.4 ± 8.4	24.8 ± 3.7	30.9 ± 3.7	34.9 ± 2.4	39.3 ± 2.3	47.3 ± 6.2
CRF, treadmill (mL⋅kg^−1^⋅min^−1^)	43.2 ± 12.3	28.4 ± 3.4	35.7 ± 2.4	41.7 ± 2.3	47.9 ± 2.9	62.2 ± 8.4
CRF assessment mode						
Cycle ergometer [%]	29 858 [76.2]	6003 [76.8]	5839 [75.2]	6078 [76.5]	6250 [77.0]	5688 [75.3]
Treadmill [%]	9339 [23.8]	1812 [23.2]	1924 [24.8]	1872 [23.5]	1864 [23.0]	1867 [24.7]
Metabolic syndrome traits						
Waist circumference (cm)	90.4 ± 12.1	97.5 ± 13.5	92.7 ± 11.7	90.4 ± 10.5	87.3 ± 9.9	83.8 ± 10.1
Central obesity [%]	9120 [23.3]	3661 [46.8]	2306 [29.7]	1717 [21.6]	926 [11.4]	510 [6.8]
SBP (mmHg)	120.7 ± 13.1	125.1 ± 13.3	122.0 ± 13.1	120.1 ± 12.5	118.7 ± 12.6	117.5 ± 12.6
DBP (mmHg)	78.2 ± 8.6	81.0 ± 8.5	79.2 ± 8.5	78.1 ± 8.3	76.9 ± 8.5	75.6 ± 8.4
Hypertension [%]	12 269 [31.3]	3659 [46.8]	2807 [36.2]	2275 [28.6]	1998 [24.6]	1530 [20.3]
Fasting glucose (mmol⋅L^−1^)	5.4 (5.1–5.7)	5.5 (5.2–5.9)	5.4 (5.1–5.8)	5.4 (5.1–5.7)	5.3 (5.0–5.6)	5.3 (5.0–5.6)
Hyperglycaemia [%]	14 453 [36.9]	3660 [46.8]	3170 [40.8]	2838 [35.7]	2577 [31.8]	2208 [29.2]
HDL‐c (mmol⋅L^−1^)	1.5 (1.2–1.8)	1.4 (1.2–1.7)	1.4 (1.2–1.7)	1.5 (1.2–1.8)	1.5 (1.3–1.8)	1.6 (1.3–1.9)
HDL‐c (males < 1.0, females < 1.3) [%]	3350 [8.5]	984 [12.6]	754 [9.7]	669 [8.4]	550 [6.8]	393 [5.2]
Triglycerides (mmo⋅L^−1^)	1.0 (0.7–1.5)	1.2 (0.9–1.8)	1.1 (0.8–1.6)	1.0 (0.8–1.5)	0.9 (0.7–1.3)	0.8 (0.7–1.2)
Triglycerides (≥ 1.7) [%]	7475 [19.1]	2277 [29.1]	1787 [23.0]	1539 [19.4]	1122 [13.8]	750 [9.9]
MetS trait count						
0 traits [%]	13 279 [33.9]	1205 [15.4]	2006 [25.8]	2630 [33.1]	3509 [43.2]	3929 [52.0]
1 trait [%]	12 479 [31.8]	2138 [27.4]	2476 [31.9]	2807 [35.3]	2721 [33.5]	2337 [30.9]
2 traits [%]	7910 [20.2]	2164 [27.7]	1905 [24.5]	1596 [20.1]	1331 [16.4]	914 [12.1]
3 traits [%]	3948 [10.1]	1556 [19.9]	1008 [13.0]	666 [8.4]	438 [5.4]	280 [3.7]
4 traits [%]	1381 [3.5]	653 [8.4]	326 [4.2]	214 [2.7]	99 [1.2]	89 [1.2]
5 traits [%]	200 [0.5]	99 [1.3]	42 [0.5]	37 [0.5]	16 [0.2]	6 [0.1]
MetS presence (≥ 3 traits)						
No [%]	33 668 [85.9]	5507 [70.5]	6387 [82.3]	7033 [88.5]	7561 [93.2]	7180 [95.0]
Yes [%]	5529 [14.1]	2308 [29.5]	1376 [17.7]	917 [11.5]	553 [6.8]	375 [5.0]

*Note:* Data are presented as mean ± standard deviation, median (interquartile range) or number [column percentage] where appropriate.

Abbreviations: BMI, body mass index; CRF, cardiorespiratory fitness; DBP, diastolic blood pressure; FLI, fatty liver index; HDL‐c, high‐density lipoprotein cholesterol; MetS, metabolic syndrome; SBP, systolic blood pressure; SES, socioeconomic status.

Across CRF quintiles, participants in the lowest quintile (Q1) were older and had higher BMI, body fat percentage and FLI values compared to those in the highest quintile (Q5). The number of MetS traits and prevalence of individual MetS components decreased progressively across increasing CRF quintiles. For example, central obesity was present in 46.8% of individuals in Q1 compared with 6.8% in Q5; hypertension in 46.8% versus 20.3%; and elevated triglycerides in 29.1% versus 9.9%, respectively. Overall, MetS (≥ 3 traits) was present in 14.1% of the cohort, decreasing in a stepwise fashion across quintiles from 29.5% in Q1 to 5.0% in Q5.

### 3.2 Associations Between Continuous CRF and MetS Outcomes

#### 3.2.1 Number of MetS Traits

Associations between continuous CRF and MetS outcomes are shown in Table 2. In the minimally adjusted model (Model 1), each 1 mL⋅kg^−1^⋅min^−1^ higher CRF was associated with a 2.8% lower MetS trait count (*p* < 0.001), with similar results following demographic adjustment (Model 2; −2.9%; *p* < 0.001). Further adjustment for adiposity (Model 3) and hepatic steatosis (Model 4) sequentially attenuated the magnitude of associations; however, inverse associations persisted (−1.1% and −0.6%, respectively; both *p* < 0.001).

**TABLE 2:** Associations between continuous cardiorespiratory fitness (CRF; per 1 mL⋅kg^−1^⋅min^−1^) and metabolic syndrome outcomes.

Outcome	Effect estimate (95% CI)	% change	*p*
MetS trait count (rate ratio; effect per 1 mL⋅kg^−1^⋅min^−1^ higher CRF)			
Model 1 (minimally adjusted)	0.972 (0.970–0.973)	−2.8%	< 0.001
Model 2 (demographic adjusted)	0.971 (0.970–0.973)	−2.9%	< 0.001
Model 3 (adiposity adjusted)	0.989 (0.987–0.990)	−1.1%	< 0.001
Model 4 (hepatic steatosis adjusted)	0.994 (0.992–0.996)	−0.6%	< 0.001
MetS presence (prevalence ratio; effect per 1 mL⋅kg^−1^⋅min^−1^ higher CRF)			
Model 1 (minimally adjusted)	0.942 (0.939–0.945)	−5.8%	< 0.001
Model 2 (demographic adjusted)	0.940 (0.936–0.943)	−6.0%	< 0.001
Model 3 (adiposity adjusted)	0.979 (0.976–0.982)	−2.1%	< 0.001
Model 4 (hepatic steatosis adjusted)	0.992 (0.990–0.995)	−0.8%	< 0.001

*Note:* Rate ratios for MetS trait count were obtained using negative binomial regression, and prevalence ratios for MetS presence were obtained using Poisson regression with robust standard errors. Effect estimates are expressed per 1 mL⋅kg^−1^ ⋅min^−1^ higher CRF. Rate ratios or prevalence ratios < 1.000 indicate lower MetS burden with higher CRF. Model 1 adjusted for CRF assessment mode; Model 2 additionally adjusted for age, sex, SES, assessment year and UK region of assessment; Model 3 additionally adjusted for BMI; Model 4 additionally adjusted for fatty liver index.

Abbreviations: BMI, body mass index; CI, confidence interval; CRF, cardiorespiratory fitness; MetS, metabolic syndrome; SES, socioeconomic status.

#### 3.2.2 Prevalence of MetS

Similarly, for MetS presence, each 1 mL⋅kg^−1^⋅min^−1^ higher CRF was associated with a 5.8% lower prevalence of MetS in Model 1 (*p* < 0.001; Table 2), with comparable results in Model 2 (−6.0%; *p* < 0.001). Additional adjustment for adiposity and hepatic steatosis attenuated the magnitude of associations, though inverse associations remained (−2.1% and −0.8% for Models 3 and 4, respectively; both *p* < 0.001).

#### 3.2.3 Dose–Response Relationship

Natural cubic spline analyses demonstrated inverse, graded associations between CRF and both predicted MetS trait count and predicted MetS prevalence in males and females (Figure 1). Associations were steeper at lower CRF levels and flattened at higher values, consistent with a non‐linear, dose–response relationship. Patterns were broadly similar in males and females, although absolute predicted values were lower in females.

**FIGURE 1:**
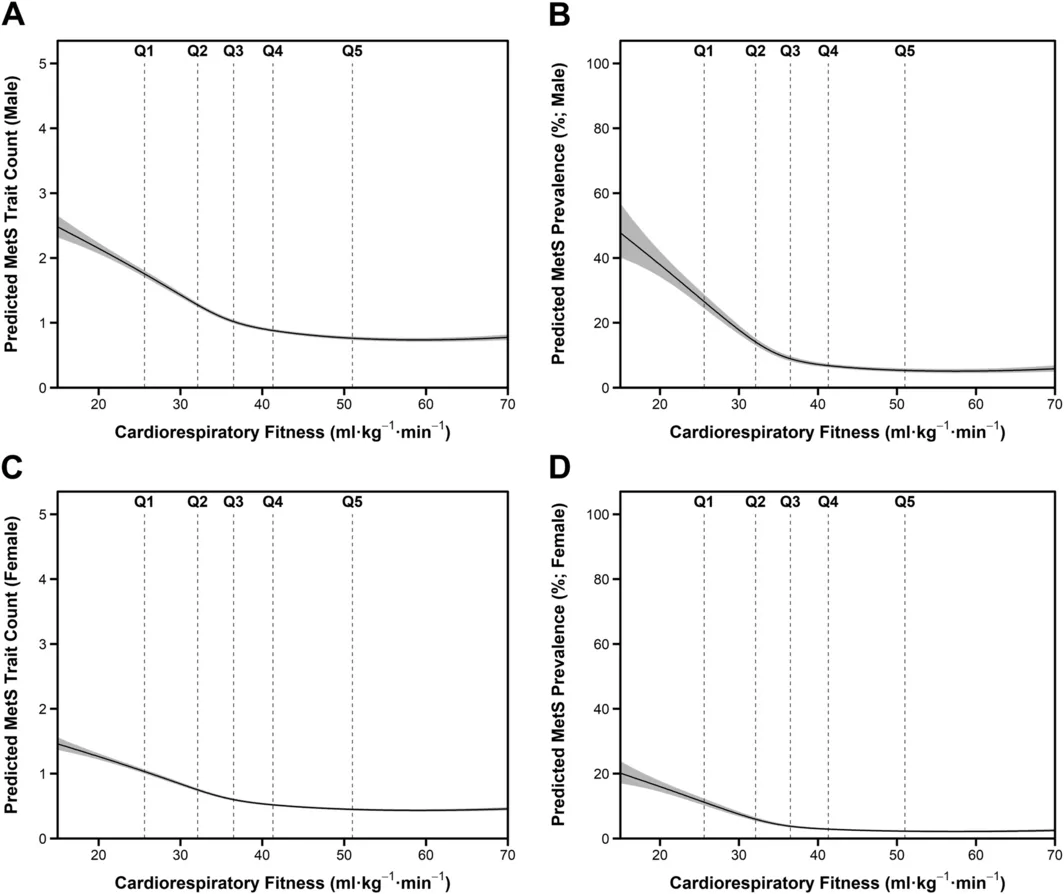
Spline‐modelled associations of CRF with predicted MetS trait count (A and C) and predicted MetS prevalence (%) (B and D) for males (A and B) and females (C and D), from demographic‐adjusted models (Model 2). CRF was modelled as a continuous exposure using natural cubic splines. Predicted values were obtained from Poisson regression models with robust standard errors and represent covariate‐adjusted estimates calculated at fixed values of confounders (median age, SES and assessment year; sex (male or female); most common category for CRF assessment mode and UK region of assessment). Shaded regions represent 95% confidence intervals (CIs). Vertical dashed lines indicate the mean CRF value within each sex‐ and mode‐specific CRF quintile (Q1–Q5). CI, confidence interval; CRF, cardiorespiratory fitness; MetS, metabolic syndrome; SES, socioeconomic status.

### 3.3 Associations Between CRF Quintiles and MetS Outcomes

#### 3.3.1 Number of MetS Traits

Associations between CRF quintiles and MetS outcomes are presented in Table 3, with demographic‐adjusted (Model 2) results illustrated in Figure 2. Compared with the highest CRF quintile (Q5), progressively lower CRF quintiles were associated with higher MetS trait count in a graded, stepwise manner (all *p* < 0.001). In Model 2, individuals in the lowest CRF quintile (Q1) had a 2.4‐fold higher MetS trait count compared with Q5. Adjustment for adiposity and hepatic steatosis sequentially attenuated effect estimates; however, the graded association persisted (all *p* < 0.001).

**TABLE 3:** Associations between cardiorespiratory fitness (CRF) quintiles and metabolic syndrome outcomes.

Outcome	Q1 (lowest CRF)		Q2		Q3		Q4		Q5 (highest CRF)
	Effect estimate (95% CI)	*p*	Effect estimate (95% CI)	*p*	Effect estimate (95% CI)	*p*	Effect estimate (95% CI)	*p*	Effect estimate (95% CI)
MetS trait count (rate ratio)									
Model 1	2.629 (2.514–2.749)	< 0.001	1.991 (1.902–2.084)	< 0.001	1.619 (1.546–1.696)	< 0.001	1.252 (1.194–1.313)	< 0.001	1.00 (ref)
Model 2	2.419 (2.309–2.534)	< 0.001	1.885 (1.800–1.975)	< 0.001	1.573 (1.501–1.649)	< 0.001	1.228 (1.170–1.288)	< 0.001	1.00 (ref)
Model 3	1.452 (1.381–1.527)	< 0.001	1.380 (1.314–1.448)	< 0.001	1.278 (1.218–1.342)	< 0.001	1.126 (1.072–1.183)	< 0.001	1.00 (ref)
Model 4	1.235 (1.173–1.300)	< 0.001	1.191 (1.134–1.252)	< 0.001	1.154 (1.099–1.213)	< 0.001	1.090 (1.037–1.145)	< 0.001	1.00 (ref)
MetS presence (prevalence ratio)									
Model 1	5.995 (5.405–6.650)	< 0.001	3.570 (3.201–3.981)	< 0.001	2.337 (2.083–2.622)	< 0.001	1.385 (1.221–1.571)	< 0.001	1.00 (ref)
Model 2	5.386 (4.838–5.995)	< 0.001	3.310 (2.965–3.695)	< 0.001	2.198 (1.959–2.466)	< 0.001	1.337 (1.179–1.516)	< 0.001	1.00 (ref)
Model 3	2.224 (1.998–2.476)	< 0.001	2.082 (1.875–2.312)	< 0.001	1.672 (1.500–1.863)	< 0.001	1.185 (1.053–1.334)	< 0.001	1.00 (ref)
Model 4	1.317 (1.202–1.442)	< 0.001	1.230 (1.122–1.348)	< 0.001	1.159 (1.053–1.276)	< 0.001	1.062 (0.957–1.179)	0.257	1.00 (ref)

*Note:* Rate ratios for metabolic syndrome trait count were estimated using negative binomial regression, and prevalence ratios for MetS presence were estimated using Poisson regression with robust standard errors. Effect estimates compare each CRF quintile with the highest CRF quintile, which serves as the reference category. Rate ratios or prevalence ratios > 1.000 indicate higher metabolic syndrome burden compared with the highest CRF quintile. Model 1 adjusted for CRF assessment mode (minimally adjusted); Model 2 additionally adjusted for age, sex, SES, assessment year, UK region of assessment (demographic adjusted); Model 3 additionally adjusted for BMI (adiposity adjusted); Model 4 additionally adjusted for fatty liver index (hepatic steatosis adjusted).

Abbreviations: BMI, body mass index; CI, confidence interval; CRF, cardiorespiratory fitness; MetS, metabolic syndrome; SES, socioeconomic status.

**FIGURE 2:**
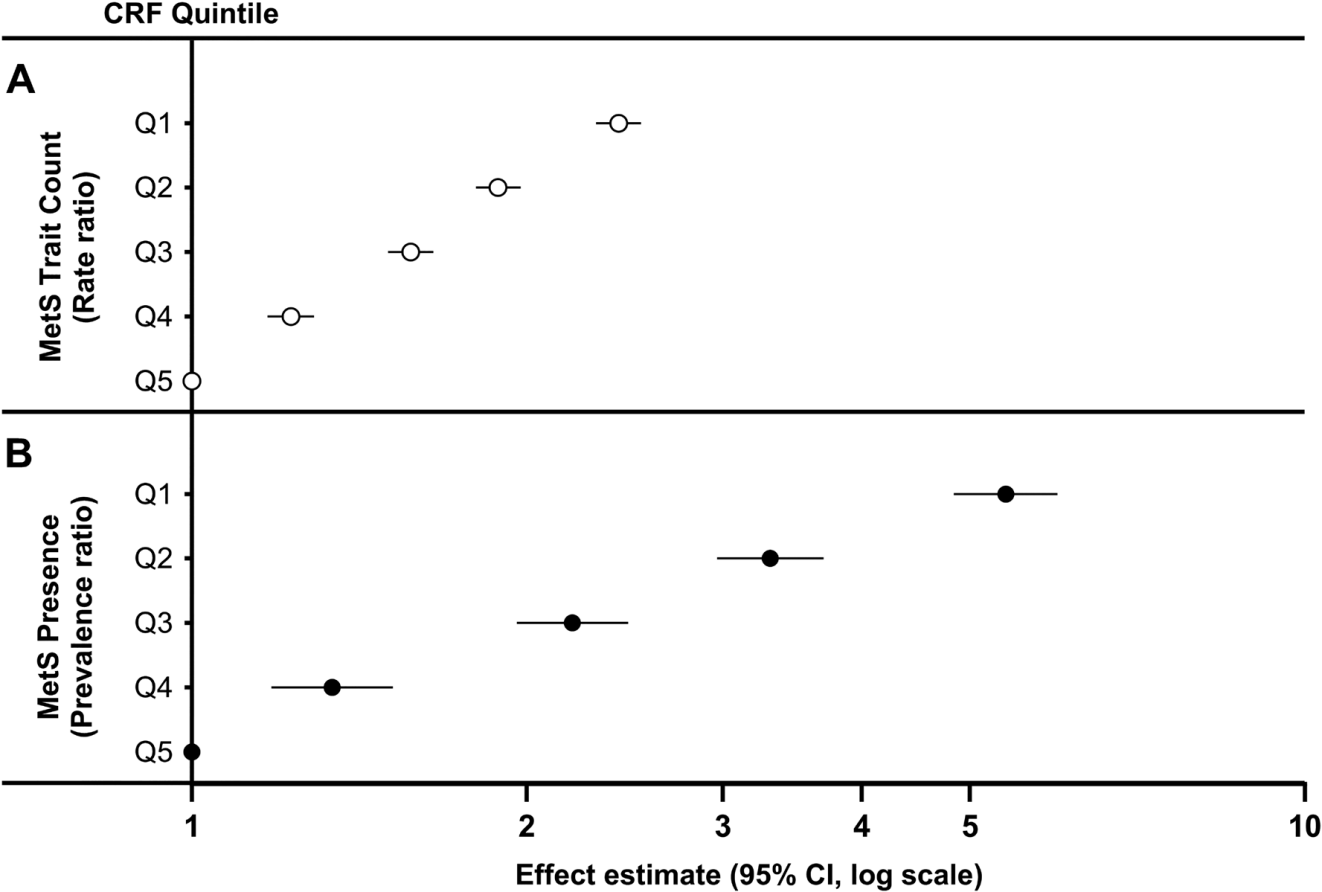
Forest plot of the associations of CRF quintiles with MetS trait count (A) and MetS presence (B) from demographic‐adjusted models (Model 2). Data are presented as rate ratios with 95% CIs for MetS trait count and prevalence ratios with 95% CIs for MetS presence. Estimates compare each CRF quintile (Q1–Q4) with the reference quintile (Q5), where Q1 corresponds to the lowest CRF group and Q5 corresponds to the highest CRF group. Models were adjusted for CRF assessment mode, age, sex, SES, assessment year and UK region of assessment. The *x*‐axis is displayed on a logarithmic scale. CI, confidence interval; CRF, cardiorespiratory fitness; MetS, metabolic syndrome; SES, socioeconomic status.

#### 3.3.2 Prevalence of MetS

Similarly, lower CRF quintiles were associated with progressively higher MetS presence (Table 3 and Figure 2B). In the demographic‐adjusted model (Model 2), individuals in Q1 had a 5.4‐fold higher MetS prevalence compared with Q5, with a stepwise gradient across quintiles (all *p* < 0.001). Although adjustment for adiposity and hepatic steatosis attenuated the magnitude of associations, lower CRF quintiles remained associated with higher MetS presence, particularly in Q1–Q3 (all *p* < 0.001).

### 3.4 Associations Between Continuous CRF and Individual MetS Components

Associations between continuous CRF and individual MetS components from the demographic‐adjusted model (Model 2) are presented in Table S2. The strongest association was for central obesity, with each 1 mL⋅kg^−1^⋅min^−1^ higher CRF associated with 6.6% lower prevalence (*p* < 0.001). Inverse associations were also observed for hypertension (−2.0%), hyperglycaemia (−1.0%), low HDL‐c (−4.0%) and elevated triglycerides (−3.6%) per 1 mL⋅kg^−1^⋅min^−1^ higher CRF (all *p* < 0.001).

### 3.5 Sensitivity Analyses

Sensitivity analyses substituting body fat percentage for BMI in adiposity‐ (Model 3) and hepatic steatosis‐adjusted (Model 4) models (*n* = 38 955) can be found in Tables S3 (continuous CRF) and S4 (CRF quintiles). The overall pattern, magnitude and interpretation of associations were materially unchanged compared with the primary analyses.

## 4 Discussion

In this large cross‐sectional study of 39 197 UK adults, higher CRF was associated with both lower MetS trait count and lower prevalence of established MetS. These relationships were strongest at lower CRF levels and appeared to flatten at higher CRF levels. Furthermore, these associations were broadly consistent across sexes and remained evident, although attenuated, after adjustment for total and liver‐specific adiposity. Together, these findings extend existing literature by showing that CRF is associated not only with binary MetS presence, but also with the cumulative burden of its individual traits. They also suggest that differences in MetS burden are greatest at the lower end of the CRF distribution. Given the high and rising prevalence of MetS and related diseases, the clinical and public health importance of translating these findings into more targeted and individualised exercise prescription and prevention strategies is considerable.

Previous studies have consistently shown that CRF is inversely associated with the prevalence and incidence of MetS [12, 13, 14], including graded associations across fitness levels in women [37] and prospective associations with MetS development in men and women [19, 38]. These findings have been observed using both directly measured VO_2_max [37] and maximal exercise test‐derived estimates of CRF [19, 38]. Our findings are consistent with this earlier work but extend it by characterising the association between CRF and cumulative MetS trait burden across a substantially larger cohort of both males and females. By contrast, trait count captures the accumulation of discrete metabolic abnormalities in a clinically interpretable manner that is more granular than a binary threshold. For context, meta‐analytic evidence from randomised controlled trials suggests that 12 weeks of exercise training can increase CRF by approximately 4 mL⋅kg^−1^⋅min^−1^ [39]. In the present cross‐sectional dataset, a 4 mL⋅kg^−1^⋅min^−1^ difference in CRF would be associated with approximately 11% lower MetS trait count and 22% lower MetS prevalence. Nevertheless, these estimates reflect between‐person associations and should not be interpreted as the expected effects of increasing CRF by this amount. Given that risk increases incrementally as metabolic abnormalities accumulate, rather than only once a diagnostic threshold is reached [2, 40], these findings suggest that CRF is relevant across the full spectrum of metabolic risk and support its value not only as an integrative marker of metabolic health, but also as a clinically meaningful vital sign and prevention target [17, 20, 41].

A notable finding was the non‐linear nature of the association between CRF and MetS outcomes. Across spline and quintile analyses, these inverse relationships were strongest at lower fitness levels (Q1–Q3), with visual inspection of the spline curves suggesting a flattening of the associations at approximately 43 mL⋅kg^−1^⋅min^−1^ in men and 37 mL⋅kg^−1^⋅min^−1^ in women. While these should not be interpreted as distinct thresholds, at higher CRF values, comparatively smaller differences in predicted MetS trait burden and prevalence were observed. Accordingly, relative to the lowest fitness quintile, the greatest difference in MetS burden was among individuals with modestly higher CRF, whereas differences across higher fitness quintiles were comparatively smaller. This pattern was reflected in descriptive prevalence estimates, where the absolute difference in MetS prevalence was markedly greater between Q1 and Q2 than between Q4 and Q5 (29.5% vs. 17.7%, compared with 6.8% vs. 5.0%). Taken together, these findings suggest that the most substantial differences in metabolic health are evident at relatively modest CRF levels, supporting the hypothesis that achievable improvements in fitness may translate into meaningful reductions in metabolic risk at the population level; however, this requires confirmation in prospective and experimental studies. The apparent flattening of the association at higher CRF levels also raises the possibility that variation in metabolic health among fitter individuals may be more strongly related to other determinants, including genetic predisposition and modifiable lifestyle factors that were beyond the scope of the present study.

The pattern of association was broadly similar in males and females, indicating that higher CRF was associated with a more favourable metabolic profile in both sexes [12, 13, 17, 41]. Notably, this comparison has been underexplored, as several previous studies examined predominantly, or exclusively, male cohorts, limiting understanding of potential sex‐specific differences. Although the shape of association was comparable between sexes, females had lower predicted MetS trait count and prevalence across the CRF range. Furthermore, the inverse association with predicted MetS burden at low CRF values appeared less steep in females. This may reflect underlying behavioural and biological differences in metabolic risk, including sex‐related variation in body fat distribution, insulin sensitivity and lipid metabolism [42, 43]. Given the relatively young mean age of the cohort, many female participants were likely to have been premenopausal, and the known protective metabolic effects of oestrogen may have contributed to their lower absolute burden [44, 45]. Nevertheless, the consistency of the inverse association across sexes supports the public health relevance of CRF to metabolic health. Future studies should also examine whether these associations differ across age groups or life stages, given the well‐established age‐related changes in CRF and metabolic risk.

Another key finding was that the inverse association between CRF and MetS outcomes remained evident after BMI and FLI adjustment, although effect estimates were attenuated. This extends previous work, which has largely adjusted only for demographic and clinical factors [12, 15, 18, 19], and aligns with a recent meta‐analysis demonstrating that CRF predicts all‐cause and cardiovascular mortality independently of BMI status [46]. Therefore, our findings suggest that the association between CRF and MetS burden is partly, but not entirely, accounted for by total adiposity and liver‐specific ectopic fat. However, the FLI‐adjusted analyses should be considered exploratory and interpreted with caution given that FLI incorporates waist circumference and triglycerides, which are themselves components of the MetS outcomes. The additional attenuation observed after FLI adjustment should therefore be confirmed in studies using more direct measures of hepatic steatosis, such as magnetic resonance imaging.

The persistence of these associations suggests that CRF may capture aspects of metabolic health beyond adiposity alone. Although the cross‐sectional design precludes causal or mechanistic inference, several physiological pathways may contribute. Higher CRF has been linked to greater insulin sensitivity, enhanced skeletal muscle oxidative capacity, improved glucose disposal and more favourable lipid metabolism [47]. Additionally, it is associated with lower systemic inflammation and improved vascular function, both of which may contribute to a healthier metabolic profile [48]. These observations are consistent with the possibility that CRF captures aspects of metabolic health not fully reflected by adiposity‐related measures, reinforcing CRF as an important and modifiable correlate of metabolic health.

To further understand which aspects of MetS were most strongly associated with CRF, we analysed each MetS component separately. Higher CRF was most strongly associated with lower central obesity prevalence, followed by favourable lipid profiles, whereas associations with blood pressure and fasting glucose were more modest. This suggests that the overall relationship between CRF and metabolic burden may particularly reflect differences in adiposity and lipid metabolism, relative to glycaemic regulation [18]. The prominence of central obesity is consistent with the attenuation after adjustment for BMI, body fat percentage and FLI, and supports adiposity as a key correlate of both lower fitness and greater metabolic dysfunction. This interpretation is biologically plausible, given evidence that exercise training can preferentially reduce visceral adipose tissue relative to body weight loss, thereby exerting favourable effects on central adiposity [49]. The persistence of inverse associations with HDL and triglycerides is notable and may be consistent with mechanisms not fully accounted for by adiposity, potentially reflecting differences in skeletal muscle metabolism and lipid handling [18]. Collectively, these findings suggest that CRF is associated with multiple components of metabolic health, although the strength of association varies across traits.

This study has several strengths, including a large sample of both males and females, standardised exercise‐based estimation of CRF and robust assessment of multiple cardiometabolic biomarkers and adiposity‐related covariates. Furthermore, the analysis moves beyond conventional binary MetS definitions by examining trait count as a clinically interpretable index of cumulative metabolic burden. However, several limitations should be acknowledged. First, the cross‐sectional design precludes causal inference and raises the possibility of reverse causality. Second, CRF was estimated from submaximal exercise testing rather than directly measured by maximal cardiopulmonary exercise testing. Although the assessments were based on established graded exercise protocols and prediction equations, testing within the healthcare setting was routinely terminated at 85% of age‐predicted maximal heart rate or due to other clinical termination criteria, given the diverse participant population. Consequently, our estimated CRF may underestimate the maximal aerobic capacity that would have been observed had participants continued to volitional exhaustion. Third, data on important lifestyle‐ and health‐related factors, including habitual dietary behaviours, muscular strength and other health conditions, were unavailable in the present study. Future investigations incorporating these variables will help establish whether the observed associations remain robust after more comprehensive adjustment. Finally, the data were derived from health assessments related to independent healthcare, and the cohort are likely more affluent than the wider UK population, which may limit generalisability to more socioeconomically diverse groups.

In conclusion, in this large cross‐sectional study of UK adults, higher CRF was associated with lower MetS trait count and lower MetS prevalence in a graded, non‐linear manner, with the strongest associations at lower fitness levels. These associations were evident in both sexes and persisted, although with attenuation, after adjustment for BMI and hepatic steatosis, suggesting potential relationships beyond adiposity. These findings support CRF as a clinically relevant and potentially modifiable marker of metabolic health and highlight the potential public health relevance of modest improvements in fitness, particularly among the least fit adults, as a strategy that may help reduce MetS burden even when adiposity changes are limited.

## Author Contributions

S.A.W. contributed to conceptualisation, formal analysis, visualisation and writing (original draft and review and editing) of the study. A.P. contributed to conceptualisation and writing (original draft and review and editing) of the study. K.D. contributed to conceptualisation, data curation and writing (review and editing) of the study. C.G. contributed to data curation and writing (review and editing) of the study. D.J.C. contributed to conceptualisation and writing (review and editing) of the study. S.E.K. contributed to conceptualisation and writing (review and editing) of the study. C.J.A.P. contributed to conceptualisation and writing (review and editing) of the study. J.G.S. contributed to conceptualisation and writing (review and editing) of the study. G.E. contributed to conceptualisation and writing (review and editing) of the study. A.C. contributed to conceptualisation and writing (review and editing) of the study. D.J.S. contributed to conceptualisation and writing (review and editing) of the study. G.P.A. contributed to conceptualisation and writing (review and editing) of the study. T.Y. contributed to conceptualisation and writing (review and editing) of the study. J.A.K. contributed to conceptualisation, formal analysis, visualisation and writing (original draft and review and editing) of the study. All authors have read and approved the final version of the manuscript and agree with the order of presentation of the authors.

## Funding

This work was supported by NIHR Leicester Biomedical Research Centre and NIHR Nottingham Biomedical Research Centre.

## Conflicts of Interest

K.D. and C.G. are employees of Nuffield Health. J.G.S. has received research funding from Astra Zeneca, Galectin, Kowa, Novo Nordisk, Regeneron and Zydus, and consults/advisory board for Novo Nordisk, Madrigal and Everyday Health. J.G.S. is currently employed by Astra Zeneca, but this manuscript was developed prior to employment. D.J.C. has received investigator‐initiated research funding from Novo Nordisk and Astra Zeneca, consultancy fees (paid to the University of Liverpool) from Madrigal and educational support (to attend conferences) from a variety of companies including Perspectum. S.E.K. has received honoraria for speaking engagements from Novo Nordisk. All other co‐authors declare that they have no competing interests.

## Supporting information


**Table S1:** Characteristics of participants included in and excluded from the final analytic sample.
**Table S2:** Associations between continuous cardiorespiratory fitness (CRF; per 1 mL⋅kg^−1^⋅min^−1^) and individual metabolic syndrome (MetS) traits from demographic‐adjusted models (Model 2).
**Table S3:** Sensitivity analysis of the associations between continuous cardiorespiratory fitness (CRF; per 1 mL⋅kg^−1^⋅min^−1^) and metabolic syndrome outcomes, adjusting for body fat percentage (*n* = 38 955).
**Table S4:** Sensitivity analysis of the associations between cardiorespiratory fitness (CRF) quintiles and metabolic syndrome outcomes, adjusting for body fat percentage (*n* = 38 955).
**Figure S1:** Participant flow diagram showing derivation of the final analytic sample.
**STROBE Statement**. Checklist of items that should be included in reports of observational studies.

## Data Availability

The data that support the findings of this study are not openly available due to reasons of commercial sensitivity and are available from the corresponding author upon reasonable request.
