# Supplementary material for: Higher Cardiorespiratory Fitness Is Associated With a Lower Burden of Metabolic Syndrome Traits: A Cross‐Sectional Analysis of 39 197 UK Adults
Source: Diabetes Obes Metab. 2026 Sep 17;28(12):11608–20. doi: 10.1111/dom.71367 (PMC13731045; doi:10.1111/dom.71367)
Supplement: Supplementary file 1 [file DOM-28-11608-s001.docx]

**Higher cardiorespiratory fitness is associated with a lower burden of metabolic syndrome traits: a cross-sectional analysis of 39,197 UK adults**

Scott A. Willis, Annalena Phillipp, Kevin Deighton, Christopher Goodwin, Daniel J. Cuthbertson, Shelley E. Keating, Christopher J.A. Pugh, Jonathan G. Stine, Gaël Ennequin, Alexis Couret, David J. Stensel, Guruprasad P. Aithal, Thomas Yates, James A. King

**Table of contents**

Supplementary Methods ……………………………………………………………… Page 2

Supplementary Table S1 ……………………………………………………………… Page 4

Supplementary Table S2 ……………………………………………………………… Page 7

Supplementary Table S3 ……………………………………………………………… Page 8

Supplementary Table S4 ……………………………………………………………… Page 9

Supplementary Figure S1 …………………………………………………………….. Page 10

STROBE Checklist …………………………………………………………………… Page 11

Supplementary References …………………………………………………………… Page 17

**Supplementary Methods**

*Additional details on the cardiorespiratory fitness assessments*

Participants completed a standardised graded exercise assessment using either a treadmill or cycle ergometer, performed by a health and wellbeing physiologist under physician supervision. Although based on established maximal incremental exercise protocols^1,2,3^, both assessments were implemented submaximally within the healthcare setting and routinely terminated at 85% of age-predicted maximal heart rate, or earlier according to participant or clinical termination.

*Pre-test procedures*

Before testing, participants underwent a resting 10-lead electrocardiogram (ECG) with modified limb-lead placement (clavicle and anterior superior iliac spine) to verify normal cardiac rhythm. A physician performed a cardiovascular assessment to exclude contraindications (e.g. unstable angina, uncontrolled arrythmias, recent myocardial infarction, systolic blood pressure > 160 mmHg or diastolic blood pressure > 100 mmHg). Participants were asked to limit caffeine within the 4 hours preceding the ECG and cardiovascular assessment, and to wear suitable exercise clothing.

*Treadmill protocol*

The treadmill assessment followed the multistage speed and gradient progression of the Bruce protocol^1^, beginning at 2.74 km·h⁻¹ and 10 % incline, with both speed and grade increased every 3 min. Heart rate and rhythm were monitored continuously, while blood pressure was measured during the final minute of each stage. The test was terminated at 85% of the participant’s age-predicted maximum heart rate, on participant request, or if any clinical termination criteria were met (e.g., systolic blood pressure > 220 mmHg, diastolic blood pressure > 110 mmHg, arrhythmias, angina, dizziness, or failure of heart rate/blood pressure to rise).

Estimated VO_2_peak (ml∙kg^-1^∙min^-1^), defined as the estimated oxygen uptake corresponding to the highest exercise level attained during the assessment, was derived from total exercise duration using the following sex-specific prediction equations:

Males: VO_2_ = 14.8 – (1.379 x T) + (0.451 x T^2^) – (0.012 x T^3^)^4^

Females: VO_2_ = 4.38 x T – 3.9^5^

where *T* is represents total treadmill exercise duration in minutes.

*Cycle ergometer protocol*

The cycle ergometer assessment used a multistage graded protocol, beginning at 25 W (females) or 50 W (males), with 25 W increments every 2 min while maintaining 70-80 rpm^2,3^. Workload continued to 85% of the participant’s age-predicted maximum heart, or earlier because of fatigue, participant request, or the clinical termination criteria described above.

Estimated VO_2_peak (ml∙kg^-1^∙min^-1^), defined as the estimated oxygen uptake corresponding to the highest workload attained during the assessment, was derived from final power output and body mass using the following sex-specific prediction equations:

Males: VO_2_ = (kgm·min⁻¹ × 1.9) + 260 + (3.5 × body mass [kg])^6^

Females: (kgm·min⁻¹ × 1.6) + 205 + (3.5 × body mass [kg])^7^

where power (W) × 6 = kgm·min⁻¹

**Supplementary Tables**

**Supplementary Table S1. Characteristics of participants included in and excluded from the final analytic sample.**

| **Variable** | **Included participants** | ***n*** | **Excluded participants** | ***n*** |
| --- | --- | --- | --- | --- |
|  |  |  |  |  |
| **Demographics** |  |  |  |  |
| Age (years) | 45.8 ± 9.0 | 39197 | 41.9 ± 11.3 | 591052 |
| Sex |  | 39197 |  | 591237 |
| *Female [%]* | 12331 [31.5] |  | 252263 [42.7] |  |
| *Male [%]* | 26866 [68.5] |  | 338974 [57.3] |  |
| SES (decile) | 8 (6–9) | 39197 | 7 (5–9) | 571786 |
| **Adiposity and body composition** |  |  |  |  |
| Weight (kg) | 80.7 ± 15.7 | 39197 | 78.4 ± 24.0 | 581475 |
| BMI (kg∙m^-2^) | 26.3 ± 4.1 | 39197 | 26.1 ± 4.6 | 581475 |
| Body fat (%) | 25.9 ± 7.5 | 38955 | 25.9 ± 8.5 | 522524 |
| Fatty liver index (AU) | 30.1 (11.5–60.2) | 39197 | 28.5 (10.4–60.9) | 395471 |
| **Cardiorespiratory fitness** |  |  |  |  |
| CRF (ml∙kg^-1^∙min^-1^) | 37.2 ± 10.0 | 39197 | 36.1 ± 9.7 | 77493 |
| CRF, cycle ergometer (ml∙kg^-1^∙min^-1^) | 35.4 ± 8.4 | 29858 | 35.0 ± 8.8 | 19111 |
| CRF, treadmill (ml∙kg^-1^∙min^-1^) | 43.2 ± 12.3 | 9339 | 43.9 ± 12.5 | 9600 |
| CRF assessment mode |  | 39197 |  | 53010 |
| *Cycle ergometer [%]* | 29858 [76.2] |  | 37306 [70.4] |  |
| *Treadmill [%]* | 9339 [23.8] |  | 15704 [29.6] |  |
| **Metabolic syndrome traits** |  |  |  |  |
| Waist circumference (cm) | 90.4 ± 12.1 | 39197 | 89.0 ± 13.2 | 546871 |
| Central obesity [%] | 9120 [23.3] | 39197 | 130245 [23.8] | 546871 |
| SBP (mmHg) | 120.7 ± 13.1 | 39197 | 120.1 ± 14.8 | 580430 |
| DBP (mmHg) | 78.2 ± 8.6 | 39197 | 77.0 ± 9.9 | 580430 |
| Hypertension [%] | 12269 [31.3] | 39197 | 162190 [27.4] | 580430 |
| Fasting glucose (mmol∙L^-1^) | 5.4 (5.1–5.7) | 39197 | 5.2 (4.9–5.6) | 431712 |
| Hyperglycaemia [%] | 14453 [36.9] | 39197 | 117343 [27.2] | 431712 |
| HDL-c (mmol∙L^-1^) | 1.5 (1.2–1.8) | 39197 | 1.5 (1.3–1.8) | 433384 |
| HDL-c (males < 1.0, females < 1.3) [%] | 3350 [8.5] | 39197 | 40649 [9.4] | 433384 |
| Triglycerides (mmol∙L^-1^) | 1.0 (0.7–1.5) | 39197 | 1.0 (0.7–1.5) | 433354 |
| Triglycerides (≥ 1.7) [%] | 7475 [19.1] | 39197 | 84905 [19.5] | 433354 |
| MetS trait count |  | 39197 |  | 396336 |
| *0 traits [%]* | 13279 [33.9] |  | 148455 [37.5] |  |
| *1 trait [%]* | 12479 [31.8] |  | 117844 [29.7] |  |
| *2 traits [%]* | 7910 [20.2] |  | 75227 [19.0] |  |
| *3 traits [%]* | 3948 [10.1] |  | 38462 [9.7] |  |
| *4 traits [%]* | 1381 [3.5] |  | 14002 [3.5] |  |
| *5 traits [%]* | 200 [0.5] |  | 2346 [0.6] |  |
| MetS presence (≥ 3 traits) |  | 39197 |  | 396336 |
| *No [%]* | 33668 [85.9] |  | 341526 [86.2] |  |
| *Yes [%]* | 5529 [14.1] |  | 54810 [13.8] |  |

Excluded participants were defined as all individuals removed during derivation of the final analytic sample. Characteristics were summarised using available valid data for each variable; therefore, denominators vary across rows due to different exclusion reasons. Implausible values were treated as missing for the relevant characteristic. Data are presented as mean ± standard deviation, median (interquartile range), or number [percentage of participants with available data for that characteristic] where appropriate. BMI, body mass index; CRF, cardiorespiratory fitness; DBP, diastolic blood pressure; HDL-c, high-density lipoprotein cholesterol; MetS, metabolic syndrome; SBP, systolic blood pressure; SES, socioeconomic status.

| **MetS trait**  **(prevalence ratio; effect per 1 ml∙kg^-1^∙min^-1^ higher CRF)** | **Effect estimate**  **(95% CI)** | **% change** | ***P*-value** |
| --- | --- | --- | --- |
| **Central obesity** (WC ≥ 94 cm in males, ≥ 80 cm in females) | 0.934 (0.931 – 0.937) | -6.6% | <0.001 |
| **Hypertension** (SBP ≥ 130 mmHg or DBP ≥ 85 mmHg) | 0.980 (0.978 – 0.981) | -2.0% | <0.001 |
| **Hyperglycaemia** (Fasting glucose ≥ 5.6 mmol∙L^-1^) | 0.990 (0.988 – 0.991) | -1.0% | <0.001 |
| **HDL-c** (< 1.0 mmol∙L^-1^ in males, < 1.3 mmol∙L^-1^ in females) | 0.960 (0.956 – 0.965) | -4.0% | <0.001 |
| **Triglycerides** (≥ 1.7 mmol∙L^-1^) | 0.964 (0.962 – 0.967) | -3.6% | <0.001 |

**Supplementary Table S2. Associations between continuous cardiorespiratory fitness (CRF; per 1 ml∙kg^-1^∙min^-1^) and individual metabolic syndrome (MetS) traits from demographic-adjusted models (Model 2).**

Prevalence ratios and 95% CIs were estimated using Poisson regression with robust standard errors. Effect estimates represent the change in prevalence of each MetS trait per 1 ml∙kg^-1^∙min^-1^ higher CRF. Prevalence ratios < 1.000 indicate lower prevalence of the corresponding MetS component with higher CRF. Models were adjusted for CRF assessment mode, age, sex, SES, assessment year, and UK region of assessment. CI, confidence interval; CRF, cardiorespiratory fitness; DBP, diastolic blood pressure; HDL-c, high-density lipoprotein cholesterol; MetS, metabolic syndrome; SBP, systolic blood pressure; SES, socioeconomic status; WC, waist circumference.

| **Outcome** | **Effect estimate (95% CI)** | **% change** | ***P*-value** |
| --- | --- | --- | --- |
| **MetS trait count (rate ratio; effect per 1 ml∙kg^-1^∙min^-1^ higher CRF)** | | | |
| Model 3 (Adiposity adjusted) | 0.987 (0.986 – 0.989) | -1.3% | <0.001 |
| Model 4 (Hepatic steatosis adjusted) | 0.995 (0.993 – 0.996) | -0.5% | <0.001 |
| **MetS presence (prevalence ratio; effect per 1 ml∙kg^-1^∙min^-1^ higher CRF)** | | | |
| Model 3 (Adiposity adjusted) | 0.959 (0.954 – 0.963) | -4.1% | <0.001 |
| Model 4 (Hepatic steatosis adjusted) | 0.993 (0.991 – 0.996) | -0.7% | <0.001 |

**Supplementary Table S3. Sensitivity analysis of the associations between continuous cardiorespiratory fitness (CRF; per 1 ml∙kg^-1^∙min^-1^) and metabolic syndrome outcomes, adjusting for body fat percentage (*n* = 38,955).**

Rate ratios for MetS trait count were obtained using negative binomial regression, and prevalence ratios for MetS presence were obtained using Poisson regression with robust standard errors. Effect estimates are expressed per 1 ml∙kg^-1^∙min^-1^ higher CRF. Rate ratios or prevalence ratios < 1.000 indicate lower MetS burden with higher CRF. Model 3 adjusted for CRF assessment mode, age, sex, SES, assessment year, UK region of assessment, and body fat percentage; Model 4 additionally adjusted for fatty liver index. CI, confidence interval; CRF, cardiorespiratory fitness; MetS, metabolic syndrome; SES, socioeconomic status.

| **Outcome** | **Q1 (Lowest CRF)** | | **Q2** | | **Q3** | | **Q4** | | **Q5 (Highest CRF)** |
| --- | --- | --- | --- | --- | --- | --- | --- | --- | --- |
|  | **Effect estimate (95% CI)** | ***P*-value** | **Effect estimate**  **(95% CI)** | ***P*-value** | **Effect estimate**  **(95% CI)** | ***P*-value** | **Effect estimate (95% CI)** | ***P*-value** | **Effect estimate (95% CI)** |
| **MetS trait count (rate ratio)** | | | | | | | | | |
| Model 3 | 1.499 (1.425 – 1.576) | <0.001 | 1.387 (1.321 – 1.456) | <0.001 | 1.267 (1.207 – 1.331) | <0.001 | 1.112 (1.058 – 1.168) | <0.001 | 1.00 (ref) |
| Model 4 | 1.209 (1.148 – 1.273) | <0.001 | 1.174 (1.117 – 1.234) | <0.001 | 1.141 (1.085 – 1.199) | <0.001 | 1.081 (1.029 – 1.137) | <0.001 | 1.00 (ref) |
| **MetS presence (prevalence ratio)** | | | | | | | | | |
| Model 3 | 3.280 (2.863 – 3.758) | <0.001 | 2.451 (2.179 – 2.758) | <0.001 | 1.685 (1.490 – 1.905) | <0.001 | 1.133 (0.962 – 1.334) | 0.136 | 1.00 (ref) |
| Model 4 | 1.308 (1.193 – 1.435) | <0.001 | 1.254 (1.143 – 1.375) | <0.001 | 1.181 (1.073 – 1.301) | <0.001 | 1.065 (0.959 – 1.182) | 0.242 | 1.00 (ref) |

**Supplementary Table S4. Sensitivity analysis of the associations between cardiorespiratory fitness (CRF) quintiles and metabolic syndrome outcomes, adjusting for body fat percentage (*n* = 38,955).**

Rate ratios for metabolic syndrome trait count were estimated using negative binomial regression, and prevalence ratios for MetS presence were estimated using Poisson regression with robust standard errors. Effect estimates compare each CRF quintile with the highest CRF quintile, which serves as the reference category. Rate ratios or prevalence ratios > 1.000 indicate higher metabolic syndrome burden compared with the highest CRF quintile. Model 3 adjusted for CRF assessment mode, age, sex, SES, assessment year, UK region of assessment, and body fat percentage (adiposity adjusted); Model 4 additionally adjusted for fatty liver index (hepatic steatosis adjusted). CI, confidence interval; CRF, cardiorespiratory fitness; MetS, metabolic syndrome; SES,

**Initial sample**

*n* = 630,557

**Eligible fasting sample**

*n* = 392,629

**Excluded:**

- Implausible values	(*n* = 3,458)

**Sample with complete covariate data**

*n* = 42,655

**Excluded:**

- Missing covariate data	(*n* = 1,521)

**Sample with complete MetS trait data**

*n* = 44176

**Sample with CRF data**

*n* = 44,530

**Excluded:**

- Missing fasting-status data (*n* = 81,943)
- Non-fasted assessment	(*n* = 155,985)

**Excluded:**

- Missing CRF / CRF mode (*n* = 348,099)

**Excluded:**

- Missing MetS trait data (*n* = 354)

**Final analytic sample**

*n* = 39,197

**Supplementary Figure S1.** Participant flow diagram showing derivation of the final analytic sample.

**STROBE Statement—checklist of items that should be included in reports of observational studies**

|  | **Item No.** | **Recommendation** | **Page  No.** | **Relevant text from manuscript** |
| --- | --- | --- | --- | --- |
| **Title and abstract** | 1 | (*a*) Indicate the study’s design with a commonly used term in the title or the abstract | 1, 4 | Title: *“...a cross-sectional analysis of 39,197 UK adults.”* Abstract: *“This cross-sectional study analysed data…”* |
|  |  | (*b*) Provide in the abstract an informative and balanced summary of what was done and what was found | 4 | Methods, key covariates, modelling approach, main ORs for MetS outcomes, and conclusion all summarised (see full abstract). |
| **Introduction** | | | |  |
| Background/rationale | 2 | Explain the scientific background and rationale for the investigation being reported | 5, 6 | Rationale linking general MetS burden to methodological approaches (binary, trait count, etc), CRF as a modifiable risk factor, and limitations of previous studies. |
| Objectives | 3 | State specific objectives, including any prespecified hypotheses | 6 | *“…The primary aim was to investigate the relationship between CRF and MetS trait count. Secondary aims were to determine whether these associations were additionally independent of total and ectopic (liver-specific) adiposity, and to characterise the associations between CRF and individual MetS traits.”* |
| **Methods** | | | |  |
| Study design | 4 | Present key elements of study design early in the paper | 6 | Data source and study population section: *“In a cross-sectional design, this study analysed anonymised data…”* |
| Setting | 5 | Describe the setting, locations, and relevant dates, including periods of recruitment, exposure, follow-up, and data collection | 6, 7 | Data source and study population section: UK hospitals, fitness centres and corporate sites. Assessments between 2006-2024. |
| Participants | 6 | (*a*) *Cohort study*—Give the eligibility criteria, and the sources and methods of selection of participants. Describe methods of follow-up  *Case-control study*—Give the eligibility criteria, and the sources and methods of case ascertainment and control selection. Give the rationale for the choice of cases and controls  *Cross-sectional study*—Give the eligibility criteria, and the sources and methods of selection of participants | 6, 7 | Data source and study population section: Cross-sectional study, adults 18-91 y attending standardised assessments; written consent; ethics approval (Loughborough University ERSC ID: 15414). |
|  |  | (*b*) *Cohort study*—For matched studies, give matching criteria and number of exposed and unexposed  *Case-control study*—For matched studies, give matching criteria and the number of controls per case | N/A | Not a matched design. |
| Variables | 7 | Clearly define all outcomes, exposures, predictors, potential confounders, and effect modifiers. Give diagnostic criteria, if applicable | 8, 9 | Exposure: CRF (VO₂peak). Outcomes: MetS outcomes. Covariates listed (age, sex, SES, BMI, body fat percentage, FLI.). |
| Data sources/ measurement | 8* | For each variable of interest, give sources of data and details of methods of assessment (measurement). Describe comparability of assessment methods if there is more than one group | 7, 8, 9 Supplementary Methods | Standardised anthropometry; fasting bloods; Bruce treadmill or cycle ramp protocols; validated equations for VO₂peak; validated definition for MetS. |
| Bias | 9 | Describe any efforts to address potential sources of bias | 10, 11, 19, 20 | Methods – statistical analysis section, Discussion – strengths and limitations section. Addresses residual confounding, selection bias (healthier/affluent cohort), and unadjusted confounding from lifestyle factors. |
| Study size | 10 | Explain how the study size was arrived at | 6, 7 | Data source and study population section: All eligible attendees with available data included (n = 39,197); secondary analysis (no a priori sample-size calc). |

| Quantitative variables | 11 | Explain how quantitative variables were handled in the analyses. If applicable, describe which groupings were chosen and why | 8, 9, 10, 11 | Methods – statistical analysis section: CRF modelled per 1 ml·kg⁻¹·min⁻¹ and as quintiles; covariates continuous/categorical as specified; MetS defined as individual traits, counts of individual traits and binary. |
| --- | --- | --- | --- | --- |
| Statistical methods | 12 | (*a*) Describe all statistical methods, including those used to control for confounding | 10, 11 | Negative binomial and Poisson regression (Models 1–4) with sequential adjustments (demographic, BMI, FLI). |
|  |  | (*b*) Describe any methods used to examine subgroups and interactions | 11 | Participant characteristics stratified by CRF quintile (Table 1). |
|  |  | (*c*) Explain how missing data were addressed | 7, 11, 12 Supplementary Figure S1, Table S1 | Complete case analysis only as implied by *“participants with available data were included”* |
|  |  | (*d*) *Cohort study*—If applicable, explain how loss to follow-up was addressed  *Case-control study*—If applicable, explain how matching of cases and controls was addressed  *Cross-sectional study*—If applicable, describe analytical methods taking account of sampling strategy | 10, 11 | Methods – statistical analysis section: Negative binomial and Poisson models with appropriate covariate control; no complex sampling weights (clinic attendees). |
|  |  | (*e*) Describe any sensitivity analyses | 10, 15 | Methods – statistical analysis section, results – sensitivity analysis section: Substituted adjustment for BMI with adjustment for body fat percentage. |
| **Results** | | | | |
| Participants | 13* | (a) Report numbers of individuals at each stage of study—eg numbers potentially eligible, examined for eligibility, confirmed eligible, included in the study, completing follow-up, and analysed | 11, 12, Supplementary Figure S1 | Results – participant characteristics: Included n = 39,197; sensitivity cohort after exclusions n reduced by 204. |
|  |  | (b) Give reasons for non-participation at each stage | 11, 12, Supplementary Figure S1 | Secondary analysis of attendees; reasons for non-attendance not available in dataset. |
|  |  | (c) Consider use of a flow diagram | Supplementary Figure S2 | Secondary analysis of attendees; reasons for non-attendance not available in dataset. |
| Descriptive data | 14* | (a) Give characteristics of study participants (eg demographic, clinical, social) and information on exposures and potential confounders | 11, 12, 27, 28, 29 | Results – participant characteristics. Full characteristics data also shown in Table 1. |
|  |  | (b) Indicate number of participants with missing data for each variable of interest | N/A | Complete-case analysis only |
|  |  | (c) *Cohort study*—Summarise follow-up time (eg, average and total amount) | N/A | Cross-sectional study design |
| Outcome data | 15* | *Cohort study*—Report numbers of outcome events or summary measures over time | N/A | Cross-sectional study design |
|  |  | *Case-control study—*Report numbers in each exposure category, or summary measures of exposure | N/A | Cross-sectional study design |
|  |  | *Cross-sectional study—*Report numbers of outcome events or summary measures | 12, 27, 28, 29 | Results – participant characteristics. Full characteristics data also shown in Table 1. |
| Main results | 16 | (*a*) Give unadjusted estimates and, if applicable, confounder-adjusted estimates and their precision (eg, 95% confidence interval). Make clear which confounders were adjusted for and why they were included | 12, 13, 14, 15, 30, 31, 32 | Negative binomial and Poisson RRs and PRs with 95% CIs across Models 1–4; covariates listed in Methods. Full results also presented in Table 2, with Model 2 results also presented in Figures 1 and 2. |
|  |  | (*b*) Report category boundaries when continuous variables were categorized | 8, 9 | Methods – outcomes section: Thresholds for individual MetS outcome listed. |
|  |  | (*c*) If relevant, consider translating estimates of relative risk into absolute risk for a meaningful time period | N/A | Cross-sectional odds across categories; absolute risks over time not applicable. |

| Other analyses | 17 | Report other analyses done—eg analyses of subgroups and interactions, and sensitivity analyses | 15 | Results section – sensitivity analysis section substituting adjustment for BMI with adjustment for body fat percentage. |
| --- | --- | --- | --- | --- |
| **Discussion** | | | | |
| Key results | 18 | Summarise key results with reference to study objectives | 15, 16 | Discussion paragraph 1: *“higher CRF was associated with both a lower MetS trait count and a lower prevalence of established MetS…”* |
| Limitations | 19 | Discuss limitations of the study, taking into account sources of potential bias or imprecision. Discuss both direction and magnitude of any potential bias | 19, 20 | Discussion paragraph 8: Cross-sectional (no causality), residual confounding, CRF estimation, selection bias toward healthier/affluent participants. |
| Interpretation | 20 | Give a cautious overall interpretation of results considering objectives, limitations, multiplicity of analyses, results from similar studies, and other relevant evidence | 15, 16, 17, 18, 19, 20 | Integrates findings with prior evidence; biological plausibility; clinical implications; call for longitudinal/interventional work. |
| Generalisability | 21 | Discuss the generalisability (external validity) of the study results | 19, 20 | Discussion paragraph 8: Notes limited generalisability due to healthier, affluent sample compared with UK population. |
| **Other information** | |  | | |
| Funding | 22 | Give the source of funding and the role of the funders for the present study and, if applicable, for the original study on which the present article is based | 20 | *“Supported by the NIHR Leicester and Nottingham Biomedical Research Centres…”* (no funder role in analysis/interpretation). |

*Give information separately for cases and controls in case-control studies and, if applicable, for exposed and unexposed groups in cohort and cross-sectional studies.

**Note:** An Explanation and Elaboration article discusses each checklist item and gives methodological background and published examples of transparent reporting. The STROBE checklist is best used in conjunction with this article (freely available on the Web sites of PLoS Medicine at http://www.plosmedicine.org/, Annals of Internal Medicine at http://www.annals.org/, and Epidemiology at http://www.epidem.com/). Information on the STROBE Initiative is available at www.strobe-statement.org

**Supplementary References**

1.	Bruce RA, Kusumi F, Hosmer D. Maximal oxygen intake and nomographic assessment of functional aerobic impairment in cardiovascular disease. *Am Heart J*. 1973;85(4):546-562.

2.	Lehmann G, Schmid S, Ammer R, Schömig A, Alt E. Evaluation of a new treadmill exercise protocol. *Chest*. 1997;112(1):98-106.

3.	Fletcher GF, Balady GJ, Amsterdam EA, et al. Exercise standards for testing and training: a statement for healthcare professionals from the American Heart Association. *Circulation*. 2001;104(14):1694-1740.

4.	Foster C, Jackson AS, Pollock ML, et al. Generalized equations for predicting functional capacity from treadmill performance. *Am Heart J*. 1984;107(6):1229-1234.

5.	Pollock ML, Foster C, Schmidt D, Hellman C, Linnerud AC, Ward A. Comparative analysis of physiologic responses to three different maximal graded exercise test protocols in healthy women. *Am Heart J*. 1982;103(3):363-373.

6.	Latin RW, Berg KE, Smith P, Tolle R, Woodby-Brown S. Validation of a cycle ergometry equation for predicting steady-rate VO2. *Med Sci Sports Exerc*. 1993;25(8):970-974.

7.	Latin RW, Berg KE. The accuracy of the ACSM and a new cycle ergometry equation for young women. *Med Sci Sports Exerc*. 1994;26(5):642-646.
